# Pharmacogenetic-Guided Antidepressant Prescribing in Adolescents (PGx-GAP): Study Protocol for a Randomized Controlled Trial

**DOI:** 10.3390/jpm16020125

**Published:** 2026-02-22

**Authors:** Meagan Shields, Laina McAusland, Madison Heintz, Katherine Rittenbach, Ross Tsuyuki, Adrian Box, Jon Emery, Jennifer Zwicker, Paul Arnold, Amanda Newton, Chad Bousman

**Affiliations:** 1 Alberta Children’s Hospital Research Institute, University of Calgary, #293 HRMB 3330 Hospital Dr. NW, Calgary, AB T2N 4N1, Canada; meagan.shields@ucalgary.ca (M.S.); paul.arnold@ucalgary.ca (P.A.); 2 Department of Medical Genetics, Cumming School of Medicine, University of Calgary, 3330 Hospital Dr. NW, Calgary, AB T2N 4N2, Canada; laina.mcausland@ucalgary.ca (L.M.); madison.heintz@ucalgary.ca (M.H.); 3 The Mathison Centre for Mental Health Research & Education, Hotchkiss Brain Institute, Cumming School of Medicine, University of Calgary, TRW 4th Floor, 3280 Hospital Dr. NW, Calgary, AB T2N 4Z6, Canada; katherine.rittenbach@ucalgary.ca; 4 Department of Psychiatry, University of Calgary, 2500 University Dr. NW, Calgary, AB T2N 1N4, Canada; 5 Department of Medicine, EPICORE Centre, Faculty of Medicine & Dentistry, University of Alberta, #362 HMRC, Edmonton, AB T6G 2S2, Canada; rtsuyuki@ualberta.ca; 6 Department of Cardiology, Faculty of Medicine & Dentistry, University of Alberta, 13–103 Clinical Sciences Building, 11304-83 Ave NW, Edmonton, AB T6G 2B7, Canada; 7 Alberta Precision Laboratories, 3520 Research Way NW, Calgary, AB T2L 2K5, Canada; adrian.box@albertaprecisionlabs.ca; 8 Population and Global Health, Lee Kong Chian School of Medicine, Nanyang Technological University, 11 Mandalay Rd, Singapore 308232, Singapore; jonathan.emery@ntu.edu.sg; 9 Department of General Practice and Primary Care, University of Melbourne, Grattan St., Parkville, VIC 3010, Australia; 10 School of Public Policy, University of Calgary, 906 8 Ave SW 5th floor, Calgary, AB T2P 1H9, Canada; zwicker1@ucalgary.ca; 11 Department of Pediatrics, Faculty of Medicine & Dentistry, University of Alberta, ECHA, 11405-87 Ave, Edmonton, AB T6G 1C9, Canada; an6@ualberta.ca

**Keywords:** pharmacogenetics, pharmacogenomics, mental health, psychiatry, depression, anxiety, selective serotonin reuptake inhibitors, antidepressants, pediatrics, adolescents

## Abstract

**Background**: Treating depression and anxiety in adolescents can be challenging due to interindividual variability in medication response. With current trial-and-error prescribing practices, adolescents may undergo multiple medication changes over months or years before an effective and tolerated drug and dose are identified. Pharmacogenomic (PGx) testing can identify interindividual differences in drug metabolism, and evidence supporting PGx-guided prescribing in adults with mental disorders is growing. However, its impact on pediatric psychotropic prescribing remains underexplored. **Methods**: This is a protocol for a parallel-arm, multicentre, randomized controlled trial. Canadian adolescents aged 12–17 years who are initiating or switching a selective serotonin reuptake inhibitor (SSRI) for depression and/or an anxiety disorder under physician care are eligible. A total of 452 participants will be randomized 1:1 to PGx-guided SSRI prescribing (experimental) or SSRI prescribing based on current practice guidelines (control). Participants, caregivers, prescribing clinicians, outcome assessors, and investigators will be blinded to treatment allocation. Dual primary outcomes are symptom remission at 12 weeks, measured with the Quick Inventory of Depressive Symptomatology–Adolescent (QIDS-A17-SR) and the Screen for Child Anxiety Related Disorders (SCARED). Secondary outcomes, assessed at 4, 8, and 12 weeks, include participant- and physician-rated changes in depressive and anxiety symptoms, role functioning, health-related quality of life, health care utilization, cost-effectiveness, side-effect burden, medication burden, and adherence. Multiple testing will be addressed using the Hochberg method, and a parallel gated analysis will account for non-actionable genotypes. Secondary analysis will estimate minimal clinically important differences for symptom and role-functioning change with PGx-guided therapy. **Discussion**: At the time of writing, 36 participants have consented and been randomized to an intervention. This trial will evaluate whether PGx-guided prescribing improves symptom remission in adolescents treated with SSRIs. If efficacious, results should be interpreted with existing pediatric pharmacokinetic, observational, and adult trial data to inform PGx use in managing pediatric anxiety and depressive disorders.

## 1. Introduction

Depression and anxiety disorders rank among the leading causes of nonfatal disability in adolescents aged 15–19 years [1] and have been correlated with lower educational attainment [2,3], reduced school attendance [4], risk of substance use [5,6], and suicidal ideation or attempt [4,7]. Current treatment guidelines for these mental disorders include psychotherapy and serotonin reuptake inhibitor (SSRI) medications [8,9,10]. With the exception of paroxetine, which has been associated with increased suicidality among youth [11,12], there is uncertainty in selection and dosing among the remaining SSRI options (citalopram, escitalopram, fluoxetine, fluvoxamine, sertraline) for adolescents.

Approximately one-half to two-thirds of people may not attain remission with medication treatment of mental disorders [13,14,15,16,17], and nearly half may experience adverse reactions [17,18,19,20]. These interindividual differences in treatment response and tolerability, combined with the availability of multiple SSRIs with comparable profiles, contribute to a ‘trial-and-error’ approach to prescribing. Such an approach often entails patients undergoing multiple sequential trials—typically lasting 4–8 weeks for depression and up to 12 weeks for anxiety disorders—before an effective and tolerated medication is found [15,21,22]. This is discouraging for patients and inefficient.

Pharmacogenetic (PGx) testing may reduce time taken to attain remission of depression and anxiety disorders by using genetically predicted metabolism to inform medication selection and dosing. The Clinical Pharmacogenetics Implementation Consortium (CPIC) has published guidelines for PGx-guided prescribing of SSRIs using CYP2B6, CYP2C19, and CYP2D6 genotypes, drawn from pharmacokinetic, observational, and clinical trial data [23]. Adults who have received PGx-guided antidepressant prescribing are 41% more likely to achieve symptom remission compared to those who receive usual care [24]. It has been suggested that PGx-guided benefits observed in adults may extend to younger populations [23,25,26,27,28,29]; however, few clinical trials have tested this proposal. One trial, designed to test PGx-guided prescribing for adolescents with generalized, separation, or social anxiety disorders, is currently active [30]. In a second, completed trial among adolescents with depression, no differences in symptom improvement, remission, or adverse events were detected between those who received PGx-guided prescribing compared to those who received usual prescribing practices [31]. These findings, however, are constrained by several methodological limitations, including the absence of prescriber blinding and insufficient statistical power [32]. The trial’s authors also note that the testing may be most beneficial for those with significant gene–drug interactions [31], citing related results from an adult trial, and recommend that future analyses account for this. We designed a trial to build on existing efforts and address previous methodological limitations.

## 2. Materials and Methods

This is a prospective, multicentre, triple-blind (participant/caregiver, prescriber, and data analyst) randomized controlled trial (RCT) using a two-arm, parallel, superiority design with a 1:1 allocation ratio. The primary objective of this trial is to evaluate the effects of PGx-guided SSRI prescribing, compared to current practice guidelines, on achieving depression and/or anxiety disorder remission in adolescents after 12 weeks of medication treatment. The trial is being conducted out of primary care (outpatient, community) settings across Western Canada. The final list of all trial locations will be included in primary results publication, aggregated if needed for participant confidentiality. Enrolment commenced in March 2025 and is projected to continue for 5 years. This trial protocol follows The SPIRIT (Standard Protocol Items: Recommendations for Interventional Trials) 2025 statement [33], with items listed Supplementary Materials, Table S1. Trial Registration: clinicaltrials.gov, Clinical trial ID: NCT06853587, Registration date: 3 March 2025, Protocol Version 3. Trial Sponsor: University of Calgary, Calgary, Alberta, Canada.

### 2.1. Public and Patient Engagement

This trial engages patient partners (a 12-member youth advisory committee) according to the International Association for Public Participation (IAP2) spectrum levels of “Inform” and “Collaborate [34].” Members are between the ages of 16 to 24 years old when recruited and have lived experience with mental disorders. The committee meets at least twice per year virtually and communicates offline as needed. Advisors are compensated for their time and expertise according to guidelines established by the Canadian Institutes of Health Research [35]. To date, committee members have provided feedback on study surveys (“Inform”) and developed trial recruitment strategies (“Collaborate”). The latter has included advertisement design, participant communication templates edits, and coordination with other patient networks. Following data analysis, members will also guide results interpretation and the results dissemination strategy (“Inform”).

### 2.2. Participants

#### 2.2.1. Inclusion and Exclusion Criteria

Adolescents are eligible to participate if they are 12–17 years old, diagnosed with depression and/or an anxiety disorder, and are starting or switching SSRI medication under the care of a primary care physician or nurse practitioner that agrees to receive study prescribing reports. They are ineligible if they have: (1) no English-language fluency, (2) co-occurring diagnosis of obsessive–compulsive disorder, psychosis bipolar disorder, eating disorder, autism spectrum disorder, fetal alcohol spectrum disorder, or intellectual disability; (3) history of non-response and/or adverse effects leading to cessation of 3 or more SSRIs; (4) participant-reported history of PGx testing of CYP2B6, CYP2C19, or CYP2D6; (5) liver or hematopoietic cell transplant; or (6) initiation of brain stimulation therapy less than 8 weeks before, or anytime during, trial participation.

#### 2.2.2. Recruitment and Informed Consent

Potential participants may be identified and referred to the trial team by a family physician, psychiatrist, nurse practitioner, pediatrician, or pharmacist. Self-referral can occur (i.e., following contact via posters, social media, institutional webpages), but the individual’s medication prescriber must approve the referral. All interested individuals will be provided with a written and verbal explanation of the trial purpose, procedures, potential risks, and benefits by the graduate research assistant (M.S.). Study eligibility is verbally confirmed with the participant or their caregiver during this conversation.

Assent is provided by interested and eligible participants aged 12 and 13 years with informed consent from their caregiver, and mature minor consent is provided by participants aged 14–17 years after determining capacity to consent. During consent/assent, participants may opt-in to future use of de-identified genetic and research data and/or to be contacted for future research opportunities.

#### 2.2.3. Discontinuation and Withdrawal

Participants may withdraw for any reason during trial participation by notifying the study team. Investigators or healthcare providers may remove the participant if they do not adhere to the study protocol, if continued participation is no longer in their best interest, or if they develop a condition that would have excluded them from the study. The reason for participant discontinuation or withdrawal will be recorded and reported in aggregate in primary results publications. Unblinding will occur in these cases if requested by the treating physician or determined appropriate by the investigators.

### 2.3. Interventions

#### 2.3.1. Pharmacogenetic Testing

All participants submit a saliva sample using an Oragene-DNA saliva kit (OG-600; DNA Genotek, Inc., Ottawa, ON, Canada) or OCR-100 ORAcollect DNA sample kit (OCR-100; DNA Genotek, Inc., Ottawa, ON, Canada) after enrolment. Alberta Precision Laboratories (Alberta Health Services, Calgary, AB, Canada) is provided with the extracted DNA to perform PGx assays for CYP2B6, CYP2D6, and CYP2C19 using the VeriDose Core 2.0 Panel and CYP2D6 CNV Panel (Agena Bioscience, San Diego, CA, USA) [36]. Samples are collected by participants at home, with instructions in multiple formats (text, infographic, and video) emailed to participants prior to mailing collection kits to their address. Participants are provided with pre-addressed return envelopes to return samples for testing. These results are used to generate the PGx-guided prescribing report.

#### 2.3.2. Experimental Intervention: PGx-Guided SSRI Prescribing

The PGx-guided prescribing report is based on CPIC’s SSRI guideline for CYP2D6, CYP2C19, and CYP2B6 genotypes (Supplementary Materials, File S1) [23]. The report is tailored to the participant with their PGx results and current medications entered into the Sequence2Script tool [37]. The tool generates a report detailing actionable recommendations for initial, titration, and maximum doses for the SSRIs citalopram, escitalopram, fluoxetine, fluvoxamine, and sertraline based on metabolism phenotypes (poor, intermediate, normal, rapid, ultra-rapid) according to CPIC guidelines. The tool also adjusts for concomitant use of medications that inhibit or induce CYP2D6, CYP2C19, or CYP2B6 enzyme function [37]. If a specific SSRI is not recommended for the participant’s PGx phenotype it is not included in the report. There is no CPIC guideline for fluoxetine at this time, so the report contains dosing recommendations according to the Guidelines for Adolescent Depression in Primary Care (GLAD-PC) [15]. If a participant is identified as having a non-actionable phenotype for an SSRI, dosage and use recommendations are based on GLAD-PC. The GLAD-PC guidelines are also used for dosing for anxiety disorders as there are no equivalent guidelines for SSRI initial, titration, and maximum dosing in pediatrics. Although GLAD-PC includes paroxetine dosing recommendations, we will not provide dosing information for this medication in the report due to its association with increased risk of suicidality in children and adolescents [11].

#### 2.3.3. Control Intervention: Non-PGx SSRI Prescribing

The non-PGx prescribing report is formatted identically to the PGx report and based on GLAD-PC recommended starting, titrating, and maximum doses for citalopram, escitalopram, fluoxetine, fluvoxamine, and sertraline [15], but its content is not informed by the PGx testing results (Supplementatry Materials, File S2). To account for some medications not being recommended for certain genotypes for the PGx-guided prescribing report, the non-PGx reports are randomized to include 3, 4, or 5 SSRIs at proportions expected in the experimental group (3.7%, 0.5%, and 95.8%, respectively). This is intended to prevent unblinding of prescribers of multiple study participants.

#### 2.3.4. Concomitant Interventions

Participants may take concomitant mental health treatments except for brain stimulation therapy, which is specified in the trial’s exclusion criteria.

#### 2.3.5. Report Delivery

The unblinded trial coordinator (M.H.) sends the participant and their medication prescriber the prescribing report based on their allocation. Prescribers are directed to use the report at their discretion, alongside other patient characteristics in prescribing decisions. Participants are reminded to arrange a medication appointment with their prescribing physician when their report is expected to arrive (i.e., 10 business days from the time the saliva sample is received) and are instructed to consult with them before making any medication changes.

#### 2.3.6. Post-Trial Care

Following participation, all adolescents and their medication prescribers are sent a complete, unblinded pharmacogenetic report generated by Sequence2Script. These reports include the participant’s genotype, phenotype, and phenoconverted phenotype (when applicable), and medication recommendations for all those with guidance currently available. Medications without CPIC recommendations will have recommendations by DPWG and/or FDA, if available. Ongoing prescribing decisions continue at the discretion of the participant and their prescriber who are provided with contact information for the Alberta Health Services’ Clinical Pharmacology Physician Consultation Service, if assistance is needed to interpret PGx results.

#### 2.3.7. Intervention Allocation and Concealment

Participants are randomized to the interventions by a third-party biostatistician not involved with the trial who uses a computer-generated sequence without restrictions. Participant allocation is only revealed to the trial coordinator to facilitate report delivery. Allocation is concealed from all other research team members as well as participants, caregivers, and medication prescribers. Blinding is facilitated by identical formatting of medication reports used in the intervention administered by an unblinded research coordinator (M.H.), and by collection of saliva samples for PGx testing from all participants regardless of assignment. In the event of participant withdrawal or after study completion, the participant or their physician may request disclosure of their intervention assignment to facilitate ongoing care.

### 2.4. Outcomes

#### 2.4.1. Primary Outcomes

Dual primary outcomes are depression and/or anxiety disorder symptom remission at 12 weeks defined as a score < 6 on the Quick Inventory of Depressive Symptomatology, Adolescent Version (QIDS-A17-SR; sensitivity = 0.38, specificity = 1.00) [38] and a score < 25 on the a Screen for Child Anxiety Related Disorders (SCARED; sensitivity = 0.71, specificity = 0.61 to 0.71) [39,40], respectively. These instruments were selected for their validation in both parent and child sources [38], low participant burden, and sensitivity to change [41,42].

#### 2.4.2. Secondary Outcomes

1. Change in QIDS-A17-SR and SCARED scores from baseline to 4, 8, and 12 weeks post-receipt of the prescribing report.
2. Change in prescriber-reported Clinical Global Impression Severity of Illness (CGI-S) and Improvement (CGI-I) scales [43] from baseline to 12 weeks post-receipt of the report.
3. Change in adverse effect burden from baseline to 4, 8, and 12 weeks, measured with the Frequency, Intensity, Burden of Side Effects Rating (FIBSER) [44,45] and Treatment-Emergent Activation and Suicidality Assessment Profile (TEASAP) [45] scales.
4. Change in medication adherence and burden from baseline to 12 weeks, measured using the Medication Adherence Report Scale (MARS-5) [46].
5. Change in role functioning from baseline to 12 weeks, measured using WHO Disability Assessment Schedule (WHODAS 2.0) [47].
6. Change in health-related quality of life (HRQoL) from baseline to 12 weeks, measured with the EuroQoL 5 Dimension—Youth (EQ-5D-Y) or EQ-5D-3L for caregiver report [48], respectively.
7. Global rating of change perceived by participants/caregivers for symptoms and functioning measured using a Global Rating of Scale (GRCS) [49].
8. Change in health care utilization and costs from baseline to 12 weeks, measured using the Resource Use Questionnaire (RUQ) [50] and administrative data extracted for physician claims, emergency department visits, hospitalizations, and medication costs.
9. Medication prescriber indication (yes/no) of SSRI prescribing report use (intervention fidelity) and perceived patient allocation (blinding).

#### 2.4.3. Data Collection

Data are collected from participants or caregivers at baseline and 4-, 8-, and 12-week follow-up. Two weeks after receipt of the blinded SSRI prescribing report is considered t0, which ensures adequate time to enact medication changes based on the report’s recommendations. Data is collected from medication prescribers at baseline and 12 weeks. Once all enrolled participants have completed the protocol or withdrawn, administrative data linked to participants’ Personal Health Numbers are collected from the Discharge Abstract Database, National Ambulatory Care Reporting System Physician Claims, and Pharmaceutical Information Network. Administrative data to be extracted are detailed in Supplementary Materials, Table S2.

Study data is collected and managed by using REDCap electronic data capture tools hosted by Faculty of Medicine & Dentistry, University of Alberta and supported by EPICORE, Department of Medicine. REDCap (Research Electronic Data Capture) is a secure, web-based application designed to support data capture for research studies, providing: (1) an intuitive interface for validated data entry; (2) audit trails for tracking data manipulation and export procedures; (3) automated export procedures for seamless data downloads to common statistical packages; and (4) procedures for importing data from external sources [51,52]. Data may be entered by participants, caregivers, and prescribers using a unique link, or by the graduate research assistant (M.S.) during phone or video-call with the participant or their caregiver. Variables to be collected can be found in Table 1, with further detail on non-outcome variables found in Supplementary Materials, Table S3. Lastly, each new prescriber referring to the study is asked to complete a one-time demographics survey (Supplementary Materials, Table S4).

Reminders are sent to participants once weekly (up to 4) with a follow-up email or call attempt after two non-responses. A non-response is recorded if primary outcome data is not received 2 weeks after the measure due date. Partially completed surveys are accepted. Prescribers are sent 3 reminders every three days for each timepoint. A $30 e-gift card is provided to participants/caregivers providing data at each timepoint. A $10 e-gift card is offered to referring physicians for each referred participant with a completed baseline assessment, and a second $10 e-gift card is offered when the physician completes the 12-week outcome survey. The data collection schedule and trial activities are outlined in Table 1. Range checks are used for numerical values entered by participants, and 10% of surveys are reviewed for accuracy. Any potential errors in data identified during review (e.g., major change in serial measures, typos, or unclear free-text responses) are clarified with the participant and corrected manually.

### 2.5. Statistical Considerations

An intention-to-treat approach will be used for primary and secondary outcome analyses. All models will adjust for baseline symptom severity, recruitment site, fluoxetine-equivalent dose, concurrent psychotherapy, and time, including a two-way interaction between treatment group and time. Models will be further adjusted if between group differences in demographics, medication adherence, or other relevant factors are detected. No interim analyses are planned for this trial.

#### 2.5.1. Primary Analyses

The primary analyses will compare intervention groups using adjusted generalized linear (logistic) mixed model analysis on the proportion achieving remission for depression and for anxiety symptoms. This approach accounts for clustering of participants within recruitment sites and provides unbiased estimates in the presence of missing data. Both primary outcomes will follow a single-family (SSRI efficacy) parallel gated approach with Hochberg correction.

As this study has two primary outcomes in a single-family outcome group (SSRI efficacy), a Hochberg-corrected, parallel gated analysis approach [53,54,55,56] will be used. First, analyses will only compare participants with an actionable genetic variant for both primary outcomes with corrected alpha. If statistical significance is reached for either outcome in the primary family, testing will proceed similarly for the entire study sample. A gated approach will prevent dilution of the intervention effect arising from participants without actionable pharmacogenetic variants. This may also prevent false positives that could occur in those without any actionable genotypes that could incorrectly lead to a rejection of the null hypothesis. Overall, this approach is considered appropriate to preserve statistical power in the setting of a gated analysis of two primary outcomes that are likely to be positively correlated [57,58]. While this approach is less conservative than others, it is important to consider that the consequences of a Type I error are relatively minor in this context, given that the intervention is non-invasive [59].

#### 2.5.2. Secondary Analyses

Secondary analyses will involve estimating between group differences in: (a) participant/caregiver and physician reported depressive and anxiety symptom severity, (b) side-effect burden, (c) medication adherence and burden, (d) role functioning, (e) HRQoL, and (f) health care resource utilization and costs. No multiplicity adjustments will be used as these hypotheses are only intended for interpretation in context with primary outcome results [56].

The anchor-based method will also be used to calculate the minimal clinically important difference (MCID) for the QIDS-A17-SR, SCARED, and WHODAS measures among participants in the PGx-guided intervention group using GRCS scores at 12 weeks for symptoms and functioning [49].

#### 2.5.3. Subgroup and Sensitivity Analyses

Planned subgroup analyses for each primary and secondary outcome are:

1. Metabolism phenotypes for each enzyme;
2. Phenoconversion presence (any one phenotype is changed by an interacting medication);
3. Testing indication (starting vs. switching medication);
4. Depression severity at baseline (moderate vs. severe);
5. Anxiety disorder subtype (as indicated by scoring of specific items on SCARED);
6. Age category (12–14 years vs. 15–17 years);
7. Sex at birth;
8. Gender identity.

#### 2.5.4. Sensitivity Analyses Will Be Conducted for the Following:

1. Exclusion of participants with obsessive–compulsive disorder diagnosed after study enrolment, as identified by any physician claim, emergency department, or hospitalization with an applicable ICD-9 or ICD-10 code occurring within 6 months of PGx test.
2. QIDS-A17-SR cut-point adjustment to <7, as suggested by a recent exploratory analysis [38].
3. Exclusion of participants whose prescriber did not use the medication report (per protocol analysis).

#### 2.5.5. Economic Evaluation

An economic evaluation will be conducted alongside the trial to assess the cost-effectiveness of pharmacogenetic (PGx)-guided SSRI prescribing compared with guideline-based prescribing over the 12-week trial period. A decision tree will be used to model relevant clinical pathways, outcomes, and health care resource use associated with each intervention.

Costs will be estimated from the health care system perspective using participant-reported resource use from the Resource Use Questionnaire (RUQ) and linked administrative data, including physician claims, emergency department visits, hospitalizations, and medication costs (Supplementary Materials, Table S2). Mean costs per participant will be calculated for each study arm.

Effectiveness will be assessed using quality-adjusted life years (QALYs), derived from EQ-5D-Y utility scores measured at baseline and 12 weeks using an area-under-the-curve approach. Incremental cost-effectiveness ratios (ICERs) will be calculated as the difference in mean costs divided by the difference in mean QALYs between intervention groups.

Uncertainty will be assessed using probabilistic sensitivity analysis with 5000 Monte Carlo simulations. Results will be presented using cost-effectiveness acceptability curves to estimate the probability that PGx-guided prescribing is cost-effective across a range of willingness-to-pay thresholds. Deterministic sensitivity analyses will examine the impact of key parameters, including the cost of PGx testing.

#### 2.5.6. Sample Size

The primary analyses require 348 adolescents (174 per group). This sample size is based on a logistic regression model with Type I error level set at 0.025, power at 80%, and an event rate of 21% under the null hypothesis, has sufficient power to detect a treatment effect size, odds ratio, of 1.71 in symptom remission [60], between those with an actionable genotype in the two intervention groups. The effect size estimate is based on the findings of a recent meta-analysis conducted among trials comparing PGx-guided prescribing to non-gene approaches in the treatment of depression [60], and the estimate of adolescents with an actionable genotype are based on preliminary, unpublished results of another study conducted by C.B. The number of enrolled participants will be 452 adolescents (226 per group) to account for a 23% attrition rate over 12 weeks [61] and the gated analysis approach to account for non-actionable genotypes [53,54]. To ensure adequate power, additional participants may be recruited to account for withdrawals. Consented participants who are randomized but do not receive the allocated intervention may be replaced. However, randomized participants who receive a study intervention and subsequently withdraw or are discontinued from the study will not be replaced. Data collected prior to withdrawal will be included in the analysis unless the participant withdraws consent for use of their data, in which case their data will be excluded, with attrition rates reported by arm.

## 3. Trial Oversight, Monitoring, and Expected Results

### 3.1. Steering Committee

The trial steering committee comprises team members (authors of this protocol) with complementary expertise and meets via quarterly virtual meetings to oversee trial progress.

### 3.2. Data and Safety Monitoring Board (DSMB)

Data quality and safety oversight is under the direction of a DSMB composed of individuals with appropriate expertise; members are independent from the study conduct and free of conflict of interest. DSMB members meet semi-annually with the executive committee to review aggregate safety, recruitment, and protocol adherence data. The DSMB may request to review efficacy, safety, and adherence data by arm in a session closed to blinded members of the research team. Following each meeting, a recommendation is provided to continue, amend, or terminate the trial, with final decisions retained by the co-principal investigators (C.B. and A.N.).

### 3.3. Adverse Event Monitoring and Reporting

Adverse events (AEs) are being identified through spontaneous report to the research team by participants, caregivers, and/or prescribers, and captured electronically through patient-reported outcome measures at 4, 8, and/or 12 weeks. The following AEs are reported to the DSMB: (1) an increase in QIDS-A17-SR of 50% or greater from baseline, (2) a score of 2 or 3 on item 13 in the QIDS-A17-SR, and (3) a score of 4–6 on item 2 or 3 of the FIBSER. The following events are considered serious AEs and will be reported to the DSMB and Research Ethics Board (REB): (1) death, (2) an emergency department visit for a suicide attempt, and (3) hospitalization. Reported events will be assessed for severity, etiology, expectedness, and relatedness [62,63].

### 3.4. Data Storage and Management

Extracted participant, caregiver, and prescriber-reported data, and all linked administrative data will be stored in a password-protected database on a secure server and a password-protected computer. Data management follows Universities of Calgary and Alberta’s Research Data Management Strategy, which is aligned with the Canadian Tri-Agency Research Data Management Policy. Data will be stored on a secure University of Alberta server for a minimum 5 years after all analyses are complete. Identifying information will be destroyed after analyses are complete; however, contact information and de-identified data will be kept indefinitely for participants who consent to be contacted for and/or have their data used in future research.

#### Biologic Specimens

DNA samples will be kept in a secure research facility at −80 °C. Genotype data will be stored separate from other participant data in password-protected databases on a secure server at the University of Calgary. Data storage and access will be under direct supervision of the PIs. At trial conclusion: (1) de-identified clinical and genotype data will be merged for data analysis, (2) PGx results will be delivered to the participants’ physicians for their medical record, and (3) biological samples from participants who did not consent to long-term storage will be destroyed.

### 3.5. Protocol Amendments

Any future protocol modifications will be submitted to the REB for approval prior to implementation, and important protocol modifications (e.g., changes to eligibility criteria, outcomes, analyses) will be added to the trial registration record and in results publications.

### 3.6. Expected Results and Dissemination Plan

This efficacy trial is expected to provide data on the impact of PGx-guided prescribing of SSRIs among adolescents with depression and anxiety. While generalizability of findings will be limited due to restrictive inclusion/exclusion criteria, these results will provide an important foundation for further real-world evaluation if this intervention shows potential benefit. Additional analyses will also improve understanding of the cost-effectiveness of PGx testing and the MCID of the QIDS-A17-SR and SCARED measures.

Upon conclusion of the trial, a summary of the results will be made available on Psychiatric Pharmacogenomics Laboratory and The Mathison Centre for Mental Health Research & Education websites with links to publications arising from the study. Dissemination will also include presentations at relevant conferences and to clinical practice groups. Publications will follow authorship guidelines established by the International Committee of Medical Journal Editors.

## 4. Discussion

At the time of writing, 36 participants have consented and been randomized to an intervention, 31 have submitted their saliva sample and received their assigned intervention, and 16 participants have completed the protocol. There have been two withdrawals prior to intervention, and three protocol deviations (missed week 12 survey).

This trial will advance pharmacogenomics and pediatric medicine by evaluating PGx-guided prescribing for adolescents with depression and anxiety. Although these disorders frequently co-occur and are often treated with the same SSRIs, no pediatric PGx trial has examined the impact of PGx-guided antidepressant prescribing while accounting for both conditions. In this study, interventions will be applied consistently, and dual primary outcomes will be defined using validated remission thresholds, regardless of the initial diagnosis. This design enables a feasible evaluation of PGx-guided SSRI prescribing in youth without requiring two separate trials. The gated analysis approach is expected to ensure that non-variant genotype prevalence does not impact measurement of intervention efficacy.

### 4.1. Highlighted Design Features

Other strengths of this trial are the selected blinding strategy and primary outcome measures. Prescriber blinding may prevent performance bias from differential cointervention use due to knowledge of intervention assignment [64,65]. While the updated CONSORT (Consolidated Standards of Reporting Trials) statement advises against the evaluation of blinding success [66], others suggest assessment of blinding integrity in psychiatric trials aids interpretability [67]. Furthermore, few PGx studies to date are blinded, possibly due the inherent difficulty of masking this intervention. If successful, other PGx trials may use similar medication reports, improving the overall quality of research in this field.

This blinding method may also reduce the risk of exaggerated intervention effects often observed with unblinded (open-label) participant-rated outcome measures (PROMs) [68,69]. The instruments used in this trial, QIDS-A17-SR [38] and SCARED [39,40], are validated parent- and child-report measures, increasing the likelihood of concordance with physician-reported measures [70,71]. Additionally, these measures are brief, sensitive to change, and can differentiate between depression and anxiety. Taken together, these methods should limit bias and facilitate accurate measurement of the true intervention effect.

### 4.2. Study Limitations

A major limitation of this trial is the reliance on prescribers to change medications according to allocation using the medication report. Although randomization and blinding mitigate inconsistent intervention delivery, low prescriber adherence may lead to an underestimated intervention effect. Sensitivity analyses planned will explore this effect; however, such findings must be interpreted alongside primary intention-to-treat analysis results—differential intervention administration may be caused by other unidentified post-randomization characteristics. Another common limitation of PGx trials are prolonged test turnaround times. Other trials have shown this results in either delaying prescribing changes and subsequent intervention effect, or simply low uptake of the intervention and decreased feasibility [72,73]. This trial will account for delayed effect with the timing of outcome measurement, and the impact of this delay can be considered when interpreting prescriber adherence results.

Another consideration in this study is the number of secondary outcomes that will be evaluated. Conflicting results between secondary outcomes with primary measures may lead to misinterpretation by readers if not properly addressed in publications. With multiple secondary outcomes, best practices include the publication of primary and secondary findings together [74], and precise interpretation of secondary findings in the context with primary outcome results [75]. Furthermore, possible dilution of intervention effects by confounders (e.g., choice of SSRI) and post-randomization characteristics (e.g., intervention use by prescribers) may lead to false-negative conclusions that would require similar care in their interpretation with the primary outcome results. This is further addressed with subgroup analyses of each primary and secondary outcome based on anticipated factors expected.

Lastly, while survey validation limits participation to those with English fluency, we acknowledge this may impact the ability to recruit equity-seeking individuals such as newcomers to Canada, and thus this trial’s generalizability to these populations.

## 5. Conclusions

This randomized controlled trial is designed to determine the efficacy of PGx-guided SSRI prescribing, compared to current practice guidelines, in the attainment of depression and anxiety remission among adolescents. Trial results will need to be interpreted with prior pediatric pharmacokinetic data, observational data, and adult trials to inform use of PGx-guided SSRI treatment in adolescents with depression and/or anxiety disorders.

## Figures and Tables

**Table 1:** Schedule of Activities.

	Study Period						
	Pre-Enrolment	Enrolment	Post-Allocation				Close-Out
Timepoint (Weeks)	−2+	−2+	0	4	8	12	13+
Eligibility:							
Eligibility screen	X						
Informed consent/assent	X						
Allocation		X					
Intervention:							
Saliva sample collection		X					
Blinded SSRI prescribing report			X				
Unblinded PGx report							X
Medication prescribing by prescriber	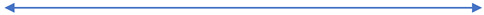						
Data Collection:							
Participant and prescriber demographics		X					
Resource use (RUQ)		X				X	
Concomitant treatment		X	X	X	X	X	
Participant or caregiver report of symptoms (QIDS-A_17_-SR and/or SCARED)		X	X	X	X	X	
Role functioning (WHODAS 2.0)		X	X	X	X	X	
Adverse events and side effects (FIBSER, TEASAP, electronic medical record)		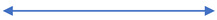					
Quality of life (EQ-5D-Y or EQ-5D-3L)		X				X	
Perceived medication effect (GRCS)						X	
Medication adherence and burden (MARS-5)		X				X	
Physician assessment of symptoms (CGI-S)		X				X	
Physician assessment of Improvement (CGI-I)						X	
Intervention fidelity and blinding						X	
Administrative health care data		X	X	X	X	X	

CGI-I: Clinical Global Impression Improvement scale; CGI-S Clinical Global Impression Severity of Illness scale; EQ-5D-Y: EuroQoL 5 Dimensions-Youth; EQ-5D-3L: EuroQoL 5-dimensional, 3 level version (caregiver report); FIBSER: Frequency, Intensity, Burden of Side Effects Rating scale; GRCS: Global Rating of Change Scale; MARS-5: Medication Adherence Report Scale; QIDS-A_17_-SR: Quick Inventory of Depressive Symptomatology, Adolescent Version; RUQ: Resource Use Questionnaire; SCARED: Screen for Child Anxiety Related Disorders; SSRI: selective serotonin reuptake inhibitor; TEASAP: Treatment-Emergent Activation and Suicidality Assessment Profile; WHODAS 2.0: World Health Organization Disability Assessment Schedule Version 2.

## Data Availability

The data presented in this study are available on request from the corresponding author due to the personal and sensitive nature of participant-level data. De-identified participant-level data and statistical code disclosure will require institutional research ethics approval for the applicable study protocol.

## Supplementary Materials

The following supporting information can be downloaded at: https://www.mdpi.com/article/10.3390/jpm16020125/s1, Table S1: SPIRIT 2025 checklist, File S1: PGx-Guided_Prescribing_Report, File S2: Non-PGx_Prescribing_Report, Table S2: Variables to be extracted for participants residing in Alberta, Canada from health administrative databases available through Alberta Health and Alberta Health Services; Table S3: Non-outcome data collected from participants or their caregivers; Table S4: Collected prescriber demographics.

## Abbreviations

The following abbreviations are used in this manuscript:

ABSPOR	Alberta Strategy for Patient-Oriented Research
AE	Adverse Event
CIHR	Canadian Institutes of Health Research
CGI-I	Clinical Global Impression-Improvement
CGI-S	Clinical Global Impression-Severity
CPIC	Clinical Pharmacogenetics Implementation Consortium
DSMB	Data and Safety Monitoring Board
ED	Emergency Department
EQ-5D-3L	EuroQoL 5 Dimensions, 3-Level (Caregiver Report)
EQ-5D-Y	EuroQoL 5 Dimensions-Youth
FIBSER	Frequency, Intensity, Burden of Side Effects Rating
GLAD-PC	Guidelines for Adolescent Depression in Primary Care
GRCS	Global Rating of Change Scale
HRQoL	Health-Related Quality of Life
ICD	International Classification of Diseases
ICER	Incremental Cost-Effectiveness Ratio
ICH-GCP	International Council for Harmonisation-Good Clinical Practice
MCID	Minimal Clinically Important Difference
MARS-5	Medication Adherence Report Scale
PGx	Pharmacogenomics/Pharmacogenetic
PI	Principal Investigator
PROM	Patient-Reported Outcome Measure
QALY	Quality-Adjusted Life Year
QIDS-A_17_-SR	Quick Inventory of Depressive Symptomatology-Adolescent Version
RCT	Randomized Controlled Trial
REDCap	Research Electronic Data Capture
REB	Research Ethics Board
RUQ	Resource Utilization Questionnaire
SAE	Serious Adverse Event
SCARED	Screen for Child Anxiety Related Disorders
SSRI	Selective Serotonin Reuptake Inhibitor
TEASAP	Treatment-Emergent Activation and Suicidality Assessment Profile
WHODAS 2.0	World Health Organization Disability Assessment Schedule 2.0
