# Supplementary material for: Pharmacogenetic-Guided Antidepressant Prescribing in Adolescents (PGx-GAP): Study Protocol for a Randomized Controlled Trial
Source: J Pers Med. 2026 Feb 22;16(2):125. doi: 10.3390/jpm16020125 (PMC12941580; doi:10.3390/jpm16020125)
Supplement: Supplementary file 1 [file jpm-16-00125-s001.zip › File S2 - Non-PGx_Prescribing_Report.pdf]

Last name, First name
DOB: Mmm DD, YYYY

Report Date
Mmm DD, YYYY

# Prescribing Report

**Patient Name**: Last, First

**Sample Type:** Saliva
**Date Collected:** Mmm DD, YYYY

Xxxxxxxx
Xxxxxxxx
Xxxxxxxx

The table below lists selective serotonin reuptake inhibitor (SSRI) options with recommended starting dosages, titration increment dosages, and maximum dosages. We recommend avoiding SSRIs not listed in the table. We encourage you to review and discuss these recommendations with the patient and family alongside possible side effects and patient preferences.

| DRUG NAME | RECOMMENDED STARTING DAILY | RECOMMENDED TITRATION | RECOMMENDED MAXIMUM DAILY |
|---|---|---|---|
| | | | |
| | | | |
| | | | |
| | | | |
| | | | |

[a] If a patient has no response at the maximum dose, consider changing to another medication.

*This report was generated as part of a clinical trial approved by the University of Calgary Conjoint Health Research Ethics Board (REB-0532). The information contained in this report is intended to be interpreted by a licensed physician or other licensed healthcare professional. The report was designed as a decision-support tool not to substitute for good clinical practice or a replacement for required medical surveillance when delivering care. The healthcare professional has ultimate responsibility for all therapeutic decisions based on the individual characteristics of the patient, of the drugs prescribed, and a comprehensive interpretation of this report.*
