# Supplementary material for: Pharmacogenetic-Guided Antidepressant Prescribing in Adolescents (PGx-GAP): Study Protocol for a Randomized Controlled Trial
Source: J Pers Med. 2026 Feb 22;16(2):125. doi: 10.3390/jpm16020125 (PMC12941580; doi:10.3390/jpm16020125)
Supplement: Supplementary file 1 [file jpm-16-00125-s001.zip › Tables S2-S4 - Data_Variables.pdf]

Data Variables

Table S2. Variables to be extracted for participants residing in Alberta, Canada from health admin-istrative databases available through Alberta Health and Alberta Health Services.

Data Source	Variable	Description
**Discharge Abstract Database (DAD) only**	Length of Stay	Number of days from admission to discharge for each unique hospitalization event. Number of days per participant may be calculated from all events for that individual.
	Intensive Care Unit (ICU) Length of Stay	Total hours spent in ICU for each unique hospitalization event.
	Case Mix Group (CMG)	Classification of each unique hospitalization event with similar clinical and resource use characteristics. CMGs are derived from Most Responsible Diagnosis, age, comorbidities, and pro-cedures.
	Resource Intensity Weight (RIW)	Relative expected resource consumption for a given CMG com-pared with the average inpatient case (RIW = 1.0). The RIW in-corporates comorbidities, interventions, age, and discharge sta-tus, and is multiplied by the provincial cost per weighted case to estimate total hospitalization cost.
**National Ambulatory Care Reporting Sys-tem (NACRS) only**	Abstract Type	Categorization of unique visit as emergency, urgent care, ad-vanced ambulatory care, and other ambulatory (e.g. outpatient clinics). Can be used to calculate average emergency depart-ment and other visits.
**DAD and NACRS**	Disposition	Categorization of participant disposition following each unique event (e.g., discharged to home, discharged to supportive liv-ing, died in facility, dead on arrival, etc.)
	Diagnosis Code	Diagnoses provided in event using ICD-10 classification. The first listed diagnosis code is the “Most Responsible Diagnosis” used in cost estimations. Up to 25 codes may be assigned in DAD, and up to 10 may be assigned in NACRS.
	Procedure Code	Interventions administered in event using Canadian Classifica-tion of Health Interventions. Up to 20 codes may be assigned in DAD, and up to 10 may be assigned in NACRS.
**Practitioner Claims**	Functional Centre Code	Type of facility where physician claim occurred (e.g., emer-gency department, physician’s office, surgical unit, etc.)
	Cost	Physician billing expense (in Canadian Dollars)
	Diagnosis Code	Diagnoses provided in event using ICD-9 classification. The first listed diagnosis code is the “Most Responsible Diagnosis” used in cost estimations. Up to 3 codes may be assigned in Claims.
**Pharmaceutical Infor-mation Network (PIN)**	Drug Identification Number (DIN)	Identifier for medication dispensed (drug, dose, formulation, brand).
	Anatomical Therapeutic Chemical (ATC) Code	Identifier for medication dispensed (drug only).
	Dispensed Amount	Total quantity of medication dispensed in the unit of the medi-cation formulation (e.g., tablets, millilitres, etc.)
	Dispensed Days’ Sup-ply	Quantity of medication dispensed, in days (based on frequency prescribed, e.g. 60 tablets for a medication prescribed twice daily would be recorded numerically as 30 for this variable)

Table S3. Non-outcome data collected from participants or their caregivers

Variable	Notes
Age	Numerical integer from 12-17 inclusive
Sex (at birth)	Single categorical response of either Male, Female, or Intersex
Gender	Categorical response (can select more than one); may specify a gender category not listed in options
Ethnicity (origin of ancestors)	Categorical response (can select more than one); may specify a gender category not listed in options
First three characters of postal code	Used to identify socioeconomic and other factors such as average household income, city/town population size, proximity to health services
Depression diagnosis characteristics (among those with depression only)	Includes age of depression diagnosis (as integer), number of lifetime depressive episodes (as integer), and date of onset of current depression episode (date, as best-estimate if precise answer not known)
Anxiety diagnosis characteristics (among those with anxiety only)	Includes age of diagnosis (as integer) and specific anxiety diagnoses provided (categorical response according to DSM-5, can select more than one)
Physical characteristics	Height and weight, as numerical data
Medications and substances	Including prior antidepressants, date, and reason for stopping; current antidepressant, other non-antidepressant psychotropics, other non-psychotropics, natural health and over-the-counter products, and substance use.
Non-medication interventions*	Services and care-providers for mental health (e.g., psychology/psychotherapy, psychiatry, social work, naturopath, etc.)

* Non-medication intervention data also includes outcome data on costs for economic analysis.

Table S4. Collected prescriber demographics

Variable	Notes
Clinic	Name of clinic and postal code
Medical Specialty	e.g., family medicine, psychiatry, pediatrician
Years of practice	Integer
Proportion of practice focused on mental health (as %)	Integer, 0-100
Proportion of adolescents in practice (as %)	Integer, 0-100
Prior experience with PGx testing	Yes or No, Dichotomous; if yes, further clarification on use of PGx previously is asked
